# Impact of Primary Diagnosis on the Outcome of Heart Transplantation in Children

**DOI:** 10.3390/jcdd12060205

**Published:** 2025-05-29

**Authors:** Csaba Vilmányi, Zsolt L. Nagy, György S. Reusz, László Ablonczy

**Affiliations:** 1 Gottsegen National Cardiovascular Center, Pediatric Heart Center, Haller Str. 29., 1096 Budapest, Hungary; csaba.vilmanyi@gokvi.hu (C.V.); zsolt.l.nagy@gokvi.hu (Z.L.N.); laszlo.ablonczy@gokvi.hu (L.A.); 2 Pediatric Center, Bókay Street Department, Faculty of Medicine, Semmelweis University, 1083 Budapest, Hungary; 3 HUN-REN–SU Pediatrics and Nephrology Research Group, 1052 Budapest, Hungary

**Keywords:** pediatrics, heart transplantation, congenital heart disease, dilatative cardiomyopathy

## Abstract

Introduction: Pediatric heart transplantation (HTX) remains the only therapeutic option for end-stage heart failure not amenable to conventional surgical or catheter interventions. We reviewed our pediatric HTX outcomes according to primary diagnosis. Patients and Methods: Sixty-two patients underwent HTX between 01/2007 and 12/2022. Patients were divided into congenital heart disease (CHD, n = 20) and cardiomyopathy (CMP, n = 42) groups. All potential variables relevant to patient recovery and long-term survival with endpoints of retransplantation or death were analyzed. Results: CHD patients underwent HTX after significantly more multiple major cardiac surgeries per patient (2.5 [0–5]) than CMP patients (0.5 [0–2], *p* < 0.01), without notable allosensitization. Post-HTX recovery was longer in CHD (mean mechanical ventilation 7 vs. 3 days, *p* = 0.001), likely due to longer surgical time (468 vs. 375 min, *p* = 0.037). There were no significant differences in the frequency of rejections between the two groups (4/20 vs. 9/42). Midterm survival was slightly better (85/70% *p* = NS) in CMP (median follow-up 44.5 [0–177] months). Conclusion: Our study confirmed good short- and long-term outcomes of pediatric HTX in both CMP and CHD. The longer postoperative recovery in CHD did not lead to higher mortality. No higher pretransplant hypersensitization was observed, possibly explaining the lack of difference in the number and severity of rejections.

## 1. Introduction

The most common causes for a heart transplant in children, other than uncorrectable heart defect and their consequences, are by far myocardial diseases (congenital or acquired) [1]. Among patients with myocardial diseases, those with dilated cardiomyopathy are the most likely to need a heart transplant. Although hypertrophic forms represent a significant proportion of cardiomyopathies (CMP), their transplantation in children is relatively rare [2]. Patients with restrictive CMP, although they constitute a relatively small proportion of all myocardial diseases, are still reported in 10–12% of all pediatric transplants according to the literature [3]. The two groups differ not only in surgical approach but also in possible changes to their immunological status [4] as a consequence of prior surgical and percutaneous interventions. Technical difficulties may arise if the patient has had significant thoracic surgeries, even multiple times, to fix complex adhesions and correct residual abnormalities. Additionally, the development of systemic-pulmonary collaterals due to chronic cyanosis can increase hemorrhagic risk and create a pronounced shunt volume, making adequate perfusion challenging [5]. Heart transplantation in heterotaxic patients is particularly difficult due to abnormal venous return and the complexity of the vitium [6,7]. Addressing all these issues can significantly extend the surgical preparation for successful implantation and the time required for surgery. Comprehensive preoperative multidisciplinary planning is essential to minimize cold ischemic time. Machine perfusion or donation after circulatory death could help with surgical planning by removing time pressure on the surgeon [8]. However, these techniques are unfortunately not yet available in Hungary. The technical challenges of introducing mechanical circulatory support (MCS) in complex congenital heart disease (CHD) may also impact the life expectancy of this group [9]. CHD patients, despite presenting higher overall operative risks and more complicated postoperative recovery, may nonetheless perform similarly in the long term as their cardiomyopathy (CMP) counterparts [10]. One study has even shown that their ten-year survival is superior to that of the latter [10]. The present study aimed to assess the outcomes of these two transplant groups over the first 15 years of our pediatric transplantation program.

## 2. Patients and Methods

Heart transplantation (HTX) as a therapeutic modality has been available at our center since 2007. In the first 15 years, through 31 December 2022, a total of 62 transplants were performed in pediatric patients. Every transplanted child under the age of 18 was included in the study. The study was carried out with the written informed consent of our patients’ guardians. Currently, only ABO compatible transplants are allowed in Hungary, and due to legal regulations, only donation after brain death is accepted. Therefore, all our patients have had this type of transplant.

The diagnosis leading to transplantation was primary disease of the myocardium (either genetically inherited or acquired) in 42 cases (CMP group) and congenital heart disease requiring a transplant in the 20 remaining cases (CHD group). Nine patients were classified as univentricular, with patients being at different stages of palliative reconstruction. ccTGA presented the second most frequent anatomical diagnosis in the CHD group with a total of four patients (20% of all CHD patients). Of the ccTGA patients, one case underwent anatomical correction, while in the others, Ebstein-like anomalies of the tricuspid valve may have contributed to the development of early heart failure despite optimal drug treatment. The etiology of heart failure in patients undergoing heart transplantation for valvular disease or tetralogy of Fallot may be explained by multiple, prolonged CBP during previous reconstruction attempts. The follow-up was complete for all 62 patients entering our program.

The need for cardiac surgery prior to transplantation was assessed. Only major surgeries (including VAD implantation) were considered (implantation and replacement of arrhythmia devices were not considered major surgeries).

The use of blood products, cryopreserved homografts and xenografts, and other foreign materials in cardiac surgery that may have an immunomodulatory effect was reviewed.

A more specific distinction was not made for blood products; only the effective number of products was calculated. The pre-HTX immunization status was evaluated by standard panel reactive antibody screening using the CDC (complement-dependent cytotoxicity) method, with 10% being considered as the cut-off value [11].

The need for MCS and its duration, preoperative hemoglobin concentration, pre- and postoperative renal function, donor ischemic time, total cardiopulmonary bypass time, and total operative times were analyzed. Days spent on mechanical ventilation after transplantation, as well as early (0–30 days), intermediate (1–12 months), and late (beyond 1 year) mortality data, were also documented.

### 2.1. Immunosuppression, Protocol Biopsy, and Rejection Therapy

Immunosuppressive therapy consisted of tacrolimus, mycophenolate mofetil (MMF), and steroids, the latter being discontinued at the end of the 6th month after transplantation. Each patient received induction therapy with basiliximab. The tacrolimus target level during the first three months was 10 to 12 ng/mL, 8 to 10 ng/mL for the following nine months, and 6 to 9 ng/mL thereafter. The starting dose of mycophenolate was 300 mg/m^2^ twice daily, raised to the maximal tolerated doses, or 600 mg/m^2^, without defining a specific target level.

Protocol biopsies were performed above 15 kg of body weight at 2–4 weeks post-HTX, and then at 3, 6, and 12 months. Diagnostic biopsies were systematically performed in the case of suspected rejection (such as elevated donor-specific antibody (DSA), reduced ventricular function, ECG abnormalities).

A relevant rejection episode was defined as histologically ISHLT (International Society for Heart and Lung Transplantation) grade II (cellular) or immunohistochemically positive antibody-mediated (humoral) [12].

If only a histological abnormality was noted without functional consequences during a protocol biopsy, then, according to the histology, intravenous steroid pulses (methylprednisolone 10 mg/kg three times every other day) were used in the event of cellular rejection, and rituximab (375 mg/m^2^ of body surface area twice, one week apart) and IVIG (1 g/kg once) in the case of humoral rejection.

If clinical or ultrasound signs of rejection were also present, a first steroid treatment was complemented according to the histology as follows: antithymocyte globulin (ATG) (1.5 mg/kg) was used in the event of cellular rejection, and plasmapheresis was considered in addition to rituximab and IVIG for humoral rejection.

### 2.2. Ethics

The study was conducted according to the guidelines of the Declaration of Helsinki, and approved by the Hungarian Medical Research Council (BM/22988-3/2024, 3/10/2024).

## 3. Statistical Analysis

All analyses were performed using Statistical Package for Social Sciences (SPSS) software (SPSS, Chicago, IL, USA). Data are expressed as mean ± SD, median and [range], or numbers and percentages as appropriate. To assess for normality, all data were first analyzed using the Kolmogorov–Smirnov test. Categorical variables were compared using the Chi-square test or Fisher’s exact test; continuous variables were compared using the Student’s *t*-test and Mann–Whitney U test where appropriate. Between-group survival after transplantation was compared using the Kaplan–Meier survival analysis, and Cox-regression. All *p*-values were two-tailed, and *p* < 0.05 was considered statistically significant.

## 4. Results

### 4.1. Demographic Data

There was a slight female dominance in the CMP group, while boys considerably outnumbered girls in the CHD group. No other differences were identified in the demographic data of the patients belonging to the two groups. Median age at cardiac transplantation was 11.5 years (range: 2.9 months–17.5 years).

The demographic data of the patients are summarized in Table 1.

Individual diagnoses of patients are shown in Table 2.

### 4.2. Follow-Up

Patients were followed until their 18th birthday or death. No patients were lost during follow-up. The median follow-up in the CMP group was 45.5 (0–177) months, while slightly less (40 months, 0.2–117.5) in the CHD group (*p* = NS).

### 4.3. Pre-Transplantation Surgical History (Including MCS)

In the CMP group, 19 patients underwent major surgery prior to transplantation. Nearly 40% (16/42) received mechanical circulatory support devices, and one infant underwent pulmonary artery banding according to the Giessen protocol as an intermediary palliation (6).

Two other patients in the CMP group had previous cardiac surgeries (ASD and VSD closure, respectively), but these were unrelated to the underlying cause leading to transplantation, which was genetically proven to be familial dilated cardiomyopathy (DCM) in both cases.

The need for MCS in the CHD group was much lower, 10% (2/20), than in the CMP group. No significant differences were found in the duration of support between the two groups (150 ± 128 vs. 145 ± 195 days, respectively, *p* = NS).

For two patients in the CHD group, transplantation was the first major cardiac procedure. In the remaining 18 patients, 44 cardiac surgical procedures were performed prior to transplantation; thus, CHD patients underwent HTX after significantly more multiple major cardiac surgeries (defined as palliative/reconstructive operations or assist-device implantation) per patient (2.5 [0–5]) compared to CMP patients (0.5 [0–2], *p* < 0. 01), without significant allosensitization.

### 4.4. Usage of Foreign Materials

Data on pretransplant, perioperative blood consumption were available for 57 patients (17/40). Comparing red blood cell usage in the two groups showed a marked difference (13 ± 17 vs. 5.9 ± 11.3, *p* = 0.1), but it was not statistically significant. Platelet usage, however, was significantly higher in the CHD group (2.9 ± 4.6 vs. 0.6 ± 1.5, *p* = 0.006). Surgical descriptions were available for 60 patients. Nonbiological materials were used in 17/9 cases. Homografts, or homograft products, were used only in the CHD group in six cases; in two other cases, xenografts were also applied during surgery.

### 4.5. Pretransplantation Sensitization

All but one patient listed for transplantation had available panel reactive antibodies (PRA) levels in the medical files. Unfortunately, the highly sensitive Luminex single antigen test has only been available in our country since 2017, so we had to use the CDC test for nearly all our patients. Despite the large amount of blood transfusions used in both groups prior to transplantation, allosensitization was only observed in three patients (CHD/CMP: 2/1). One of these was a retransplantation after a PGF (primary graft failure) and four other major heart operations with homografts and xenografts (PRA: 21%), and one after three surgeries in which a homograft was also implanted and also required MCS (PRA: 100%).

The one in the CMP group needed long-lasting MCS (PRA: 59%).

Mean PRA was not significantly different in the two groups (7.68 (0–100%) compared to 3.14 (0–59%); *p* = 0.27)

One patient received a combination of rituximab and plasmapheresis for desensitization without success. HTX was performed successfully after a positive crossmatch. The remaining two were transplanted without any significant immunological complication.

### 4.6. Pretransplantation Organ Damage

Preoperative renal function impairment differed in the two groups. Mean creatinine clearance, calculated by the bedside Swartz formula [13], was significantly lower in the CHD group (74.3 ± 20.5 vs. 90.3 ± 21.7 *p* = 0.008), and the proportion of patients with chronic renal insufficiency (defined as creatinine clearance below 60 mL/min/m^2^) was also significantly higher (6/20 (one patient with missing data) vs. 2/41 *p* = 0.012). VAD patients (from both groups) had better pre-HTX renal function compared to non-VAD patients (GFR: 80.6 ± 28.5 vs. 96.5 ± 18.1 mL/min/m^2^ *p* = 0.04). The hemoglobin concentration was measured as a further preoperative functional parameter in all patients. CHD patients had a significantly higher hemoglobin concentration (132.2 ± 34.2 g/L vs. 114.0 ± 17.1 g/L *p* = 0.07), although the mean value of each group did not indicate significant anemia.

### 4.7. Perioperative Data

Perioperative data are summarized in Table 3. No patient required permanent pacemaker therapy. Pacemaker therapy was used in 11 cases during the early postoperative period due to predominantly low atrial activity. In four patients (three CHD, one CMP), treatment was complicated by diaphragmatic paralysis, with two cases developing during pretransplantation interventions. Postoperative mechanical circulatory support (BiVAD) was required in one case due to primary graft failure. Three patients required dialysis. Total operative and cardiopulmonary bypass times were recorded to characterize the technical complexity of the surgery. As expected, CMP patients had significantly shorter pump-run times and overall operative time, with significant differences found in mechanical ventilation requirements. No significant differences in the incidence of postoperative acute kidney injury (AKI—defined according to KDIGO guidelines) [14] were found between the two groups.

### 4.8. Rejection and Graft Failure

There were 13 rejections requiring active intervention, four in the CHD group (20%), and nine in CMP patients (21%).

A total of 12 grafts in 11 patients were lost during the follow-up. No compliance issues have been identified in the background of rejections with graft loss. One of the lost grafts was a patient transplanted with a positive crossmatch. This patient’s transplant meant 4 years of good quality of life, but due to chronic humoral rejection and lack of further options, we lost her afterward. Another patient lost his graft due to therapy-resistant humoral rejection. This patient was successfully retransplanted. There were two additional patients with graft losses due to cellular rejection. There were two perioperative deaths caused by primary graft failure. One patient was bridged by ECMO to early retransplantation without success. There was no substantial difference in graft failures between the two groups (Table 4).

Overall graft survival was 83% on follow-up, with 70% in the CHD group and 85% in the CMP group (*p* = NS).

According to our data, the predicted 1-year graft survival was 80% (CI: 64.3–99.6%) in the CHD group and 95.2% (CI: 89.0–100%) in the CMP group, while the predicted five-year survival was 80% (CI: 64.3–99.6%) vs. 87.4% (CI: 75.8–100%) respectively.

**Figure 1:**
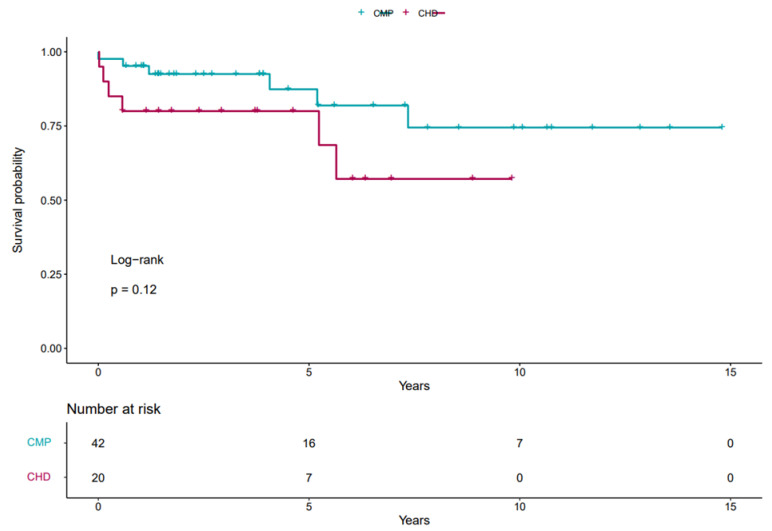
Kaplan–Meier curve of our cohort.

The Kaplan–Meier survival curves of different groups are shown in Figure 1.

Cox regression analysis showed no significant correlation between type of heart failure and mortality (with a CHD hazard ratio of 2.1 *p* = 0.2). In multivariate analysis, neither preoperative surgery, renal dysfunction, nor longer respiratory therapy had a significant effect on post-transplant mortality.

### 4.9. Neurological Outcome

Significant disability was observed after transplantation in five children due to embolic stroke. All required MCS, with the complication occurring during MCS in all but one case. There were no other notable neurological complications during and after the transplantation.

## 5. Discussion

The current study presents data related to heart transplantation in 62 children, with a special focus on etiology and management. The etiology of end-stage heart failure differs between adults and children. While primary CMP and CHD are the most common causes in pediatrics, ischemic CMP is predominant in adults [15], despite the growing adult population with congenital heart disease (ACHD). Similar to international data [16], CHD had a significant prevalence in our HTX patients. In the CHD group, Fontan failure (end-stage heart failure in patients with univentricular heart) was the most common indication for HTX. Of note, the second most common cause of CHD-related HTX was biventricular circulation with systemic right ventricle, although end-stage heart failure in this group is rather expected to occur in adulthood [17].

Impaired kidney function prior to transplantation may be a potential risk factor for post-transplant outcomes [18,19], while pretransplant VAD treatment is a known risk factor for bleeding complications and hyperimmunization [4,11]. In our patients, neither reduced pretransplant renal function nor pretransplant VAD treatment worsened patient outcomes. The former may be explained by the fact that there were no patients with severely impaired renal function or on dialysis treatment, whereas with VAD, patients were transplanted in a stable clinical condition with good organ functions, including preserved renal function. Although the preoperative hemoglobin concentration was significantly higher in the CHD group, presumably due to the compensatory polycythemia of the cyanotic patients, this was not a factor that significantly influenced the prognosis.

The number of previous major cardiac surgical procedures not only increases the technical complexity of the transplantation but also represents a significant immunological risk [20]. More than half of the 62 patients required major cardiac surgery prior to HTX (VAD or palliative/reconstructive operations). Despite this history of high incidence of major cardiac surgery, we were unable to detect increased hypersensitization in any of the groups. We cannot ignore that using the traditional complement-dependent cytotoxicity assay instead of the newer flow-cytometry crossmatching may explain the low grades of DSA [21]. However, the number of previous surgeries significantly increased both cardiopulmonary bypass and total operative times, which was mainly due to prolonged bleeding control and, particularly in the CHD group, the frequent need for additional surgical interventions.

Organ transport time and implantation time have the greatest impact on cardiac ischemic time [22,23,24], representing important factors for patient survival. Thus, an accurate surgical plan is required for complex CHD patients [25].

The most common indicators of post-transplant recovery are the number of days on mechanical ventilation, days in the intensive care unit and the total length of hospital stay [26,27]. Of the three, the number of days on ventilation is the most reliable indicator in our transplant program, since the other two indicators are significantly influenced by our protocol biopsy schedule and the patient’s and parents’ education process. Our CHD patients spent significantly longer on ventilation, which can be explained by longer operative times, related to the increased number of previous cardiac operations and additional surgical procedures during the HTX.

Severe postoperative renal dysfunction can also intuitively be considered an indicator of complicated recovery. Although this study did not show that renal dysfunction leads to a poorer prognosis, the numbers in the groups are small, and the data were not statistically significant.

Early mortality (<30 days) did not show significant intergroup differences. While primary graft failure and its complications led to early postoperative mortality, the aforementioned surgical technical challenges, the longer CPB time, and longer ventilation duration were not risk factors for early mortality in the CHD group [16].

Infection, rejection, and malignancy played a role in mortality beyond 30 days, with long-term survival slightly worse in the CHD group, although the trend was not statistically significant.

The 5-year and 10-year survival of the combined CM/CHD group is slightly lower than the recent ISHLT data [28]. This may be attributed to the relatively low-volume HTx program and the relatively small number of cases presented.

Although we can expect longer postoperative recovery in the CHD group, this did not represent a statistically significant increase in mortality, but only a trend increase. There was no difference in the degree of sensitization between the groups, which may explain the lack of difference in the number and severity of rejections. Longer term follow-up and a larger number of patients are nonetheless warranted to assess the difference in the incidence of transplant-related diseases in the two groups.

## 6. Limitations

The relatively small number of cases, the retrospective nature of the study, and the length of follow-up do not allow more definitive conclusions to be drawn, such as causal relationships or the occurrence of late complications. Unfortunately, for legal and financial reasons, patients can only be treated by a pediatrician up to the age of 18, which limits follow-up. Indeed, adding adult care data to our study would improve the follow-up results for patients transplanted during adolescence. Another shortcoming is the technique used to detect immunization; however, as noted above, single antigen testing has only been available for patients transplanted since 2017, so this was not used in this study. Preoperative proBNP could provide valuable data to better assess pre-transplant status, but in our cohort, this information was not available for all patients and could not be presented. Notwithstanding the latter, we provide important evidence that satisfactory mid- to long-term results can be achieved in both groups examined. The integration of observations and results from individual centers in this relatively small patient population, compared to studies in adults, may provide useful information for the design of further observational and controlled studies.

## Figures and Tables

**Table 1:** Demographic data of the transplant patients.

	CMP	CHD	Total
Median age at the time of HTX (years (range))	11.5 (0.2–16.9)	11.1 (0.5–17.5)	11.5 (0.21–7.5)
Male/Female	18/24	15/5	33/29
Body weight (kg)	38.4 ± 26.3	35.4 ± 21.9	37.5 ± 24.8
Height (cm)	132 ± 39.0	132 ± 38.4	132 ± 38.5
Body mass index (kg/m^2^)	18.5 ± 5.9	17.8 ± 3.6	18.3 ± 5.2
Body surface area (m^2^)	1.16 ± 0.57	1.11 ± 0.52	1.15 ± 0.55

CMP, cardiomyopathy; CHD, congenital heart disease.

**Table 2:** Pretransplant diagnosis of patients.

Diagnosis			
CHD	Number of Patients	CMP	Number of Patients
Univentricular heart, Norwood stage 1 palliation	1	Dilated CMP	33
Tricuspid atresia after pulmonary arterial banding	1	Restrictive CMP	3
Univentricular heart, Norwood stage 2 (Glenn) procedure	3	Noncompact CMP	5
Univentricular heart, Norwood stage 3 (total cavopulmonary connection)	4	Arrhythmogenic CMP	1
Tetralogy of Fallot	2		
Transposition of great arteries	1		
Congenitally corrected transposition of great arteries (CCTGA)	4		
Combined valvular aortic disease	2		
Valvular pulmonary stenosis	2		
Mean number of previous major surgeries	2.5 (0–5)		0.5 (0–2)
Mechanical circulatory support	2		16

**Table 3:** Patient data in the perioperative period.

	CMP	CHD	*p*
Ischemic time (min)	181 ± 54	208 ± 48	0.056
CPB time (min)	219 ± 127	295 ± 141	0.049
Operative time (min)	375 ± 162	468 ± 156	0.037
Ventilation (days) *	3 ± 1.9	7 ± 6.9	0.021
AKI stage 0 (n/percent)	22/52%	7/35%	0.25
AKI Stage 1 (n/percent)	6/14%	3/15%	
AKI Stage 2 (n/percent)	2/5%	3/15%	
AKI Stage 3 (n/percent)	12/29%	7/35%	
Postoperative transient pacemaker	4	7	
*Nervus phrenicus paresis*	1	3	

CHD: congenital heart disease, CMP: cardiomyopathy, CPB: cardiopulmonary bypass, AKI: acute kidney injury. * Invasive mechanical ventilation.

**Table 4:** Causes and occurrence of graft loss.

	PGF	Rejection	Infection	Tumor	Other	Early 0–30 Days	Intermediate 31–365 Days	Late +365 Days	Total
CMP N = 42	1	2	1	1	1	1	1	5	6
CHD N = 20	1	2	1	1	1	1	3	2	6

CMP: cardiomyopathy; CHD: congenital heart disease; PGF: primary graft failure.

## Data Availability

The data presented in this study are available on request from the corresponding author due to privacy reasons.

## Abbreviations

ACHD	adult congenital heart disease
AKI	acute kidney injury
ASD	atrial septal defect
ATG	antithymocyte globulin
BiVAD	biventricular assist device
ccTGA	congenitally corrected transposition of the great arteries
CDC	complement-dependent cytotoxicity
CHD	congenital heart disease
CMP	cardiomyopathy
CPB	cardiopulmonary bypass
DSA	donor-specific antibody
ECMO	extracorporeal membrane oxygenator
HTX	heart transplantation
ISHLT	International Society for Heart and Lung Transplantation
MCS	mechanical circulatory support
MMF	mycophenolate mofetil
NS	not significant
PGF	primary graft failure
VAD	ventricular assist device
VSD	ventricular septal defect
